# A Mixture of *Artemisia argyi* and *Saururus chinensis* Improves PM_2.5_-Induced Cognitive Dysfunction by Regulating Oxidative Stress and Inflammatory Response in the Lung and Brain

**DOI:** 10.3390/plants12061230

**Published:** 2023-03-08

**Authors:** Jin-Yong Kang, Jong-Min Kim, Seon-Kyeong Park, Hyo-Lim Lee, Ho-Jin Heo

**Affiliations:** 1 Division of Applied Life Science (BK21), Institute of Agriculture and Life Science, Gyeongsang National University, Jinju 52828, Republic of Korea; 2 Research and Development Division, World Institute of Kimchi, Gwangju 61755, Republic of Korea; 3 Korea Food Research institute, Wanju-Gun 55365, Republic of Korea

**Keywords:** *Artemisia argyi*, *Saururus chinensis*, fine particles, cognitive dysfunction, inflammation, oxidative stress

## Abstract

This study was performed to investigate the improving effect of a mixture of *Artemisia argyi* and *Saururus chinensis* (AASC) on cognitive dysfunction in mice with long-term exposure to fine particles (particulate matter smaller than 2.5 µm: PM_2.5_). The main compounds of AASC were identified as dicaffeoylquinic acid isomers of *A. argyi* and a quercetin-3-glucoside of *S. chinesis*. As a result of behavioral tests for the evaluation of cognitive function, it was confirmed that cognitive dysfunction was induced in the PM_2.5_ exposure group, and a tendency to improve in the AASC group was confirmed. Increased oxidative stress and inflammatory response and mitochondrial dysfunction were observed in the brain and lung tissues of the PM group. Damage to the brain and lung affected the accumulation of amyloid beta (Aβ) in the brain. It increased Aβ and induced the cholinergic dysfunction, hyperphosphorylation of the tau protein, and activation of apoptosis, leading to cognitive impairment. However, AASC suppressed brain and lung oxidative stress and inflammation, thereby suppressing brain Aβ expression. Consequently, this study shows the potential that a steady intake of plant resources with antioxidant and anti-inflammatory activity could prevent cognitive impairment caused by PM_2.5_.

## 1. Introduction

Environmental pollution is currently the most important cause of various diseases and premature death in the world, and it is estimated that more than a quarter of the causes of death are due to environmental pollution in countries with severe environmental pollution [1]. Recently, particulates, which cause air pollution, have emerged as a significant issue. Particulates are not a single substance, but a complex mixture of fine solid and liquid particles of organic and inorganic components floating in the atmosphere. Internationally, total suspended particles are divided into coarse, fine, and ultrafine according to their size, and are measured as coarse particles (PM_10_; particle diameter < 10 μm), fine particles (PM_2.5_; particle diameter < 2.5 μm), and ultrafine particles (particle diameter < 0.1 μm).

In an epidemiological study, it was confirmed that more amyloid beta (Aβ)_42_ protein is accumulated in the neurons and astrocytes of residents living in areas with severe air pollution than in those living in areas with low pollution [2]. Air pollution can lead to structural and functional changes in the brain, leading to cognitive impairment. The inhaled PM_2.5_ reaches brain tissue through systemic circulation, bypassing the blood–brain barrier (BBB), or through the olfactory bulb. The PM_2.5_ reaching brain tissue is deposited on the senile plaque, exacerbating the inflammatory response or promoting amyloid production by stimulating glial cells [3]. In addition, PM_2.5_ can enter the nasal epithelial cells and cause inflammation and brain damage. Furthermore, contaminants in PM_2.5_ that reach the alveoli without being removed by mucociliary clearance cause the production of pro-inflammatory cytokines by inducing inflammation. Accordingly, these pro-inflammatory cytokines are delivered to the brain tissue through systemic circulation, spreading nerve inflammation [4]. According to an autopsy study by Calderón-Garcidueñas et al., in people living in areas with the severe air pollution, the disruption of the BBB, endothelial activation, oxidative stress, and inflammatory cell trafficking, as well as the increment of cyclooxygenase-2 (COX-2), interleukin 1 beta (IL-1β), and a cluster of different 14 (CD14) concentrations in the olfactory bulb, frontal lobe, black matter, and vagus nerve [2], were observed. In particular, it was reported that PM_2.5_ acts as a pro-oxidant directly on lipids and proteins and promotes oxidative stress as a free radical producer [5]. Therefore, it is possible to reduce the risk of toxicity due to PM_2.5_ by sufficiently ingesting antioxidants or nutrients with anti-inflammatory action.

*Artemisia argyi* and *Saururus chinensis* are abundant in polyphenols and have various physiological activities such as antioxidant, anti-cancer, anti-inflammatory, etc. [6,7]. Recently, studies on the antioxidant and anti-inflammatory activities of *A. argyi* were reported [8,9]. It was reported that the primary polyphenols of *A. argyi* are hydroxybenzoic acids and hydroxycinnamic acids, and the primary polyphenols of *S. chinesis* are flavonoids [7,10]. Polyphenols are structurally diverse, and are abundant in fruits and vegetables. Various physiological activities of polyphenols, such as antidiabetic, antioxidant, and anti-inflammatory effects, have been reported [8]. Accordingly, a polyphenol-rich diet protects against chronic pathologies by modulating numerous physiological processes [11]. In our previous studies, it was confirmed that *A. argyi* and *S. chinesis* have a protective effect on PM_2.5_-induced cell cytotoxicity (RPMI2650, nasal cell line; A549, alveolar basal epithelial cell line; BV2, microglial cell line) (Supplementary Data, Figure S1). In addition, *A. argyi* and *S. chinesis* significantly suppress lipopolysaccharide (LPS)-induced nitric oxide (NO) production in BV2 cells (Supplementary Data, Figure S2). *S. chinesis* is more effective than *A. argyi* in suppressing NO production (Supplementary Data, Figure S2). On the other hand, *A. argyi* is more effective than *S. chinesis* on PM_2.5_-induced cell cytotoxicity, but *A. argyi* is toxic to RPMI2650 cells (>250 μg/mL) and BV2 cell (500 μg/mL) (Supplementary Data, Figure S1).

Chronic exposure to PM_2.5_ can lead to various problems because PM_2.5_ causes neuroinflammation and oxidative stress [4,5]. Therefore, it may be possible to reduce the risk of PM_2.5_ toxicity by consuming enough nutrients with antioxidant and anti-inflammatory activities. *A. argyi* has a cell protection against PM_2.5_ toxicity, and *S. chinesis* has excellent anti-inflammatory activity on microglia cells (BV2) (Supplementary Data, Figure S2). Therefore, this study was conducted to evaluate the effect of both plants with excellent anti-oxidant and anti-inflammatory activity on improving cognitive dysfunction caused by chronic exposure to PM_2.5_ [6,7].

## 2. Results and Discussion

### 2.1. Main Compounds Analysis in AASC Using UPLC/Q-TOF-MS/MS

**Figure 1:**
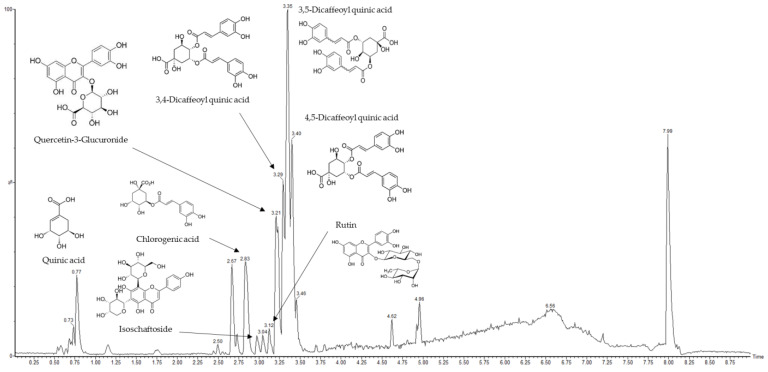
Analysis of AASC using UPLC/Q-TOF-MS/MS chromatography in negative ion mode.

The identified phenolic compounds of a mixture of *Artemisia argyi* and *Saururus chinensis* (AASC) in negative ionization mode with UPLC/Q-TOF-MS/MS analysis are as follows: quinic acid, chlorogenic acid, isoschaftoside, rutin, quercetin-3-glucuronide (Q3G), 3,4-dicaffeoyl quinic acid, 3,5-dicaffeoyl quinic acid, and 4,5-dicaffeoyl quinic acid (Table 1). Eight compounds were identified, and the dicaffeoylquinic acid (diCQA) isomers were found to be the most abundant in AASC (Figure 1). The diCQA isomers were the main compounds in *A. argyi* and quercetin-3-glucoside is the main compound in *S. chinesis* (Supplementary Data Figure S3). The compounds were analyzed using the Mass Bank database (https://massbank.eu, accessed on 1 December 2021) and previous studies [12]. The diCQAs are esters formed from quinic acid and two units of caffeic acid, and it is reported that it possesses a broad spectrum of pharmacological properties, such as anti-inflammatory and antioxidant properties [13,14]. Q3G is a primary quercetin metabolite, and diverse and interesting physiological activities of Q3G have also been reported [15,16,17,18]. AASC contains functional compounds with various physiological activities, including antioxidant and anti-inflammatory. In conclusion, it is presumed that the identified diCQA isomers and Q3G are mainly involved in the effect of PM_2.5_-induced cognitive dysfunction.

### 2.2. Evaluation of Cognitive Function with Behavioral Tests 

Air pollution can lead to structural and functional changes in the brain, leading to cognitive impairment. The epidemiological evidence proves a link between cognitive impairment and chronic exposure to PM_2.5_ [19]. 

**Figure 2:**
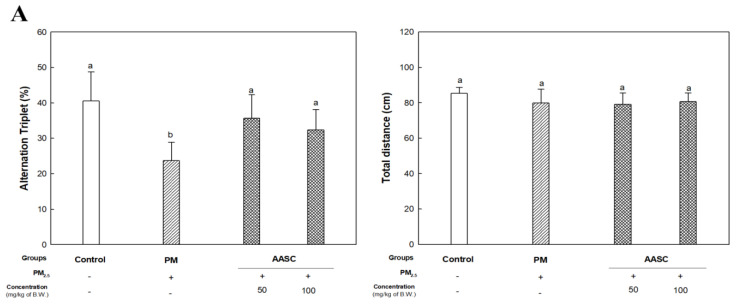

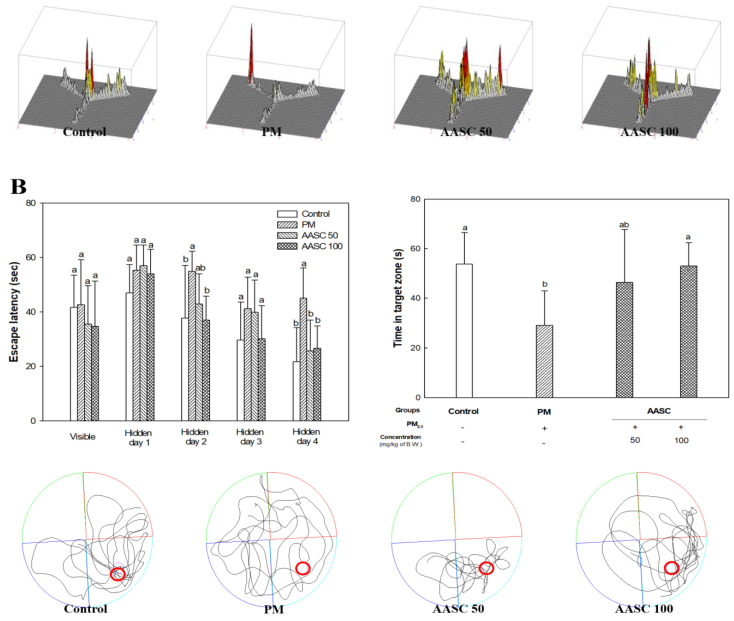
Improvement effect of AASC on cognitive dysfunction of mice exposed to PM_2.5_. (**A**) Y-maze test and (**B**) Morris water maze test. Results are indicated as mean ± SD (*n* = 9). Different small letters represent the statistical difference (*p* < 0.05) of each group in a high order.

The PM_2.5_ exposure group (PM group) showed lower cognitive function than the control group in behavioral tests (Figure 2). In a Y-maze test for the evaluation of short-term memory ability, there was no significant difference in the distance in the zone that could indicate athletic ability in all groups. However, the alternation triplet score, which can indicate short-term memory ability, decreased in the PM group compared to the control group; in contrast, there was no significant difference between the AASC groups and the control group (Figure 2A).

A Morris water maze test was performed to assess long-term memory and learning ability [20]. When the platform’s location was exposed, there was no difference between groups in time to find the platform. However, in the training period, when the platform’s position was hidden, and then the mouse was made aware of the platform’s position, the time the PM group took to recognize and find the platform did not decrease significantly compared to other groups. In a probe test where the platform was removed, and the retention time to find the area where the platform existed was evaluated, it was also confirmed that the PM group had a low retention time. On the other hand, it was confirmed that the AASC group showed improved long-term memory and learning ability through the training period and probe test compared to the PM group (Figure 2B). The results of the behavioral tests showed improved cognitive function in the AASC group. Although these results cannot be definitive evidence that AASC can improve cognitive impairment due to PM_2.5_, it is speculated that the effect of AASC can be shown indirectly.

It is estimated that oxidative stress and inflammation are the leading causes of cognitive impairment due to PM_2.5_ [3,5]. Accordingly, it is presumed that the improvement of cognitive dysfunction in the AASC group is related to the physiological activity based on the antioxidant and anti-inflammatory activity of AASC. It was already reported that *A. argyi* and *S. chinesis* have antioxidant and anti-inflammatory effects [6,7,8,9]. In addition, it was reported that diCQAs, the main compounds of *A. argyi* and the ones most abundant in AASC, and Q3G, the main compound of *S. chinesis*, have antioxidant and anti-inflammatory activities [13,14,15]. DiCQAs have particularly strong antioxidant activity because they have more hydroxyl groups than caffeoyl quinic acid [21]. It was also reported that 3,5-diCQA improves cognitive dysfunction by inhibiting oxidative damage to brain tissue [22]. As well, Kim et el., suggested that 3,5-diCQA might be a potential therapeutic for treating or preventing oxidative-stress-induced neurodegenerative diseases [23]. In addition, Q3G is reported to have enjoyable physiological activities as well as anti-inflammatory effects [16,17,18]. According to the research of Ho et al., Q3G can suppress the formation of neurotoxic oligomeric Aβ species by interrupting the initial protein–protein interaction of Aβ_1–40_ and Aβ_1–42_ [17]. In addition, it is reported that Q3G has positive effects on neurogenesis by promoting proliferation [18]. It is speculated that AASC, which contains these compounds, improves cognitive dysfunction by suppressing inflammation and increases brain oxidative stress caused by PM_2.5_.

### 2.3. Analysis of the Cholinergic System in Brain Tissue

The cholinergic system is composed of organized nerve cells and includes neurons located in the basal forebrain and their long axons that reach the cerebral cortex and the hippocampus. Acetylcholine (ACh) is used as a neurotransmitter in the transduction of action potentials and plays a critical role in hippocampus-dependent learning [24]. Cognitive impairment involves a substantial loss of the elements of the cholinergic system [25]. Dysfunction of cholinergic signaling leads to the loss of dendrites in cortical neurons; accordingly, it is observed in the late stages of Alzheimer’s disease (AD) [26].

**Figure 3:**
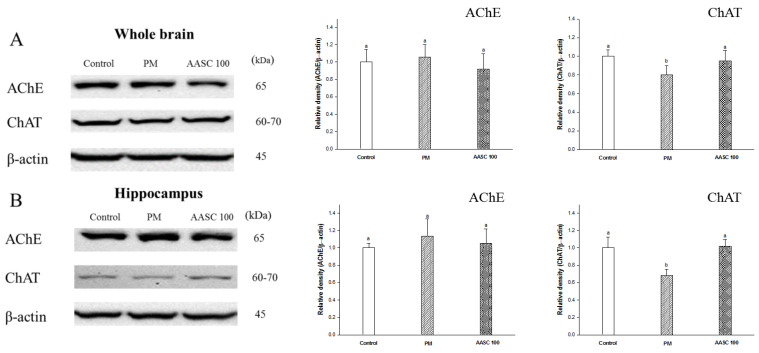
Effect of AASC on the cholinergic system in the brains of mice exposed to PM_2.5_. (**A**) Whole brain and (**B**) hippocampus. Results are indicated as mean ± SD (*n* = 9). Different small letters represent the statistical difference (*p* < 0.05) of each group in a high order.

In the PM group, a decrease in ACh levels and an increase in acetylcholinesterase (AChE) activity were observed compared with the control group. It is already reported that exposure to PM_2.5_ leads to dysregulated central nervous system (CNS) neurotransmitters [27]. On the other hand, increased ACh levels and decreased AChE activity was observed in the AASC groups (Table 2). The reduction in the AChE activity in the AASC group may be related to the AChE inhibitory activity of AASC. It was reported that the various naturally occurring compounds from plants can inhibit the enzyme AChE [28]. Research found that 3,5-diCQA, the main compound of *A. argyi* and the most abundant in AASC, inhibits AChE activity [7]. In addition, quercetin was reported to have vigorous AChE inhibitory activity [29], and its derivative, Q3G, is also expected to have AChE inhibitory activity. The elevation of ACh through AChE inhibition is often used to treat AD patients [30]. In addition, the increase in ACh contents in the AASC group is presumed to also be related to the expression change of choline acetyltransferase (ChAT). The expression of AChE was not significantly different in all groups. However, ChAT was significantly increased in the hippocampus and the whole brain of the AASC group compared to the PM group (Figure 3). It is speculated that the increased expression of ChAT may be related to the Q3G positive effect on neurogenesis [18]. The change in ChAT expression in the hippocampus should be noted. The enzyme ChAT is responsible for synthesizing ACh from acetyl CoA. A decreased ChAT level is one of the central biochemical disorders in cognitive impairment [24]. According to research by González-Castañeda et al., a decreased number of cholinergic neurons as well as decreased expression of both the ChAT gene and protein levels were confirmed in AD patients [31]. The hippocampus plays important roles in spatial memory and long-term memory, and cholinergic dysfunction significantly affects the hippocampus and its function [24]. That is, it is presumed that long-term exposure to PM_2.5_ induces neurodegenerative diseases such as AD, leading to dysfunction of cholinergic signaling. However, AASC suppressed the dysfunction of the cholinergic system caused by PM_2.5_. In other words, AASC is effective in preventing neurodegenerative diseases caused by PM_2.5_. Therefore, this result is presumed to be related to the improved cognitive function of the AASC group confirmed in the behavioral experiment (Figure 2 and Figure 3, Table 2).

### 2.4. Measurement of Oxidative Damage in the Brain, Lung Tissues, and Serum

PM_2.5_ penetrates the lungs and brain tissue, causing oxidative damage and inflammation [3,4,5]. In particular, the brain is greatly affected by oxidative stress caused by PM_2.5_ because the brain has high oxidizable polyunsaturated fatty acid and redox-active transition metal ions content [32]. An interesting fact is that oxidative stress in the brain and lungs interact with each other. Abdennour et al. proposed that the protection of both the brain and lung in brain-injured patients is necessary for the management of brain-injured patients because the brain and lung interact early and rapidly when hit by a disease process [33]. In several clinical studies, it was confirmed that lung injury occurs shortly after brain damage, and it is suggested that the harmful actions of neurotransmitters, autonomic system dysfunction, inflammation, or neurogenic pulmonary edema are the responsible mechanisms [34]. After all, in order to more effectively prevent oxidative damage to the brain caused by PM_2.5_, it is also necessary to protect lung tissue from oxidative damage. Various biochemicals were measured to confirm the oxidative damage to the lungs and brain tissue. Through these results, it was confirmed that the oxidative damage to the brain and lungs of the PM group was increased (Figure 3). The body has a complex antioxidant defense system, including superoxide dismutase (SOD), catalase, and glutathione (GSH). Antioxidant enzymes protect body tissues by suppressing oxidative damage of the vital biomolecule from the free radicals [35]. It was confirmed that increased malondialdehyde (MDA) is an indicator of oxidative stress, and decreased antioxidants, such as SOD, and reduced GSH in the lungs and brain tissue of the PM group. In contrast, these changes, such as increased MDA and decreased antioxidant enzymes in the PM group, were improved in the AASC group (Table 3). In addition, it was confirmed that the oxidative stress was increased even in the serum of the PM group. Serum lactate dehydrogenase (LDH) and ferric antioxidant power (FRAP) can indicate oxidative stress and total antioxidant capacity [36]. LDH exists in almost all the body’s tissues, and, when these tissues are damaged, they release LDH into the bloodstream. Accordingly, LDH increases under the condition of oxidative stress [37]. As a result of evaluating the serum LDH content and FRAP assay, it was confirmed that the level of LDH decreased and the FRAP activity increased in the AASC group compared with the PM group (Table 4). Ultimately, it was confirmed that the oxidative stress in the AASC group was reduced in both tissue and blood. It is presumed that the antioxidant effect of the compounds contained in *A. argyi* and *S. chinesis* suppressed oxidative stress in the brain and lung tissue. Various plants have natural antioxidants such as polyphenols, and it is reported that *A. argyi* and *S. chinesis* have excellent antioxidant activity [12,13,14]. In particular, it is reported that the diCQAs, as the main compounds of AASC, have many hydroxyl groups; accordingly, they have considerable antioxidant activity [21]. Therefore, these results show that the natural antioxidant activity of AASC effectively suppressed the oxidative stress caused by PM_2.5_ in the brain and lung tissues. In addition, it is speculated that the reduction in overall oxidative stress in both the brain and lung is closely related to the improving cognitive impairment in the AASC group.

### 2.5. Measurement of Mitochondrial Function in the Brain and Lung Tissues

The oxidative stress in cells leads to structural and functional changes in the mitochondria, and then ultimately leads to diseases by generating uncontrollable ROS [38]. In addition, the mitochondria are a key target of PM_2.5_ in both the brain and peripheral organs. PM_2.5_ contains organic compounds such as polycyclic aromatic hydrocarbons, which are more prone to accumulate in mitochondria due to the high lipid content of their membranes [38]. Accordingly, it is confirmed that the mitochondrial ROS is increased in the brain and lung tissue of the PM group with increased oxidative stress. Additionally, the levels of mitochondrial membrane potential (MMP) and ATP were decreased in the PM group (Table 5). MMP is the driving force behind ATP production; accordingly, it is a crucial indicator of mitochondrial activity. In the AASC group with reduced oxidative stress, it was confirmed that not only was the level of ROS decreased, but also that the levels of MMP and ATP increased. That is, mitochondrial dysfunction was also improved in the AASC group when compared with the PM group (Table 5). The improvement of mitochondrial dysfunction in the AASC group would be closely related to the reduction in oxidative stress in the AASC group. In addition, the improvement of mitochondrial dysfunction may be related to the monoamine oxidase (MAO) reaction. MAO, consisting of two isoforms, A and B, is a mitochondrial enzyme that produces hydrogen peroxide through MAO-catalyzed reactions in the cellular cytosol [39]. In the research of Yoshino et al., Q3G attenuated oxidative stress in brain mitochondria by interrupting the generation of hydrogen peroxide accompanying the MAO-A reaction [40]. In addition, it was reported that diCQAs can also inhibit MAO type A and B activity in neurons and astrocytes [41]. According to Gandhi and Abramov, the brain is vulnerable to damage from ROS production due to elevated MAO levels because the brain is usually exposed to high levels of monoamines [42]. It is speculated that AASC improves mitochondrial dysfunction in brain tissue by inhibiting the MAO reaction. Mitochondrial dysfunction is related to an early event in AD. There is extensive literature on the role of mitochondrial dysfunction and oxidative damage in the pathogenesis of AD; ultimately, mitochondrial dysfunction is a trigger of AD pathophysiology [43]. In addition, an exciting fact is that mitochondrial dysfunction is closely related to the disruption of the cholinergic system. According to the research of Wong et al., mitochondrial dysfunctions are connected to defective synaptic transmission and lead to changes in cholinergic and monoaminergic systems [44]. In the end, the implications of the results of mitochondrial function evaluation are clear. Ultimately, these results show that the PM_2.5_-induced increase in brain oxidative stress was suppressed by AASC, thereby improving mitochondrial dysfunction and cognitive dysfunction. 

### 2.6. Protein Expression in Lung Tissue

**Figure 4:**
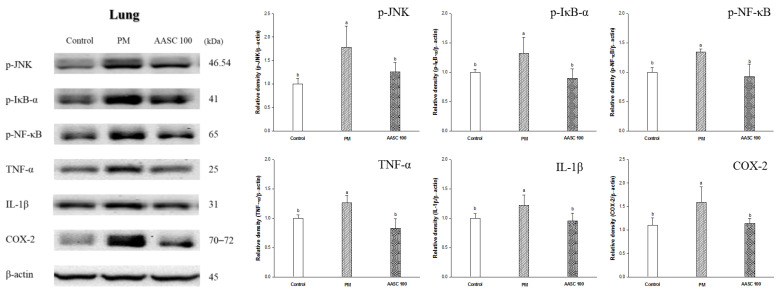
The effect of AASC on proteins related to inflammation in lung of mice exposed to PM_2.5_. Results are indicated as mean ± SD (*n* = 8). Different small letters represent the statistical difference (*p* < 0.05) of each group in a high order.

The PM_2.5_ complexes that reach the alveoli produce pro-inflammatory cytokines, inducing inflammation [4]. The changes in protein expression related to inflammation were analyzed in the lung tissue. It was confirmed that inflammation increased in the lung tissue of the PM group compared with the control group. In contrast, inflammation was improved in the AASC groups (Figure 4). The c-Jun N-terminal kinase (JNK) pathway is activated by the phosphorylation of JNK and is closely related to an inflammatory response. Activated JNK affects the maturation and activity of T cells and the synthesis of pro-inflammatory cytokines [45]. Accordingly, a decrease in JNK phosphorylation was observed in the AASC group compared to the PM group. In addition, there were results indicating that inflammation improved in the lungs of the AASC group. IκBα plays a crucial role in the inflammatory process and immune response by increasing the phosphorylation of transcription factor NF-κB, promoting the expression of cytokines, and accelerating the inflammation response by upregulating the expression of COX-2 [46]. The results of inflammation in lung tissue were observed in the AASC group and compared with the PM group (Figure 4). These results show that AASC has anti-inflammatory effects, and this result is related to the main compounds of AASC. The anti-inflammatory effect of *A. argyi* and *S. chinesis* was confirmed with cell experiments (Supplementary Data, Figure S2). The anti-inflammatory activity of diCQAs and Q3G has been reported in various studies [13,14,15,16]. 3,5-diCQA and 4,5-diCQA showed anti-inflammatory activity not only in in vitro but also in in vivo tests [13,14]. Recently, it was also reported that 3,5-diCQA suppresses inflammation-mediated pain hypersensitivity by enhancing autophagy [47]. Additionally, it has been reported that Q3G can improve inflammation by regulating inflammatory gene expression [16]. 

These results suggest that AASC-containing substances with anti-inflammatory activity are effective in ameliorating PM_2.5_-induced inflammation in lung tissue. In addition, it is presumed that the reduction in lung inflammation in the AASC group is related to improving cognitive function in the AASC group by suppressing the increase in inflammatory cytokines in brain tissue.

### 2.7. Protein Expression in Brain Tissue

**Figure 5:**
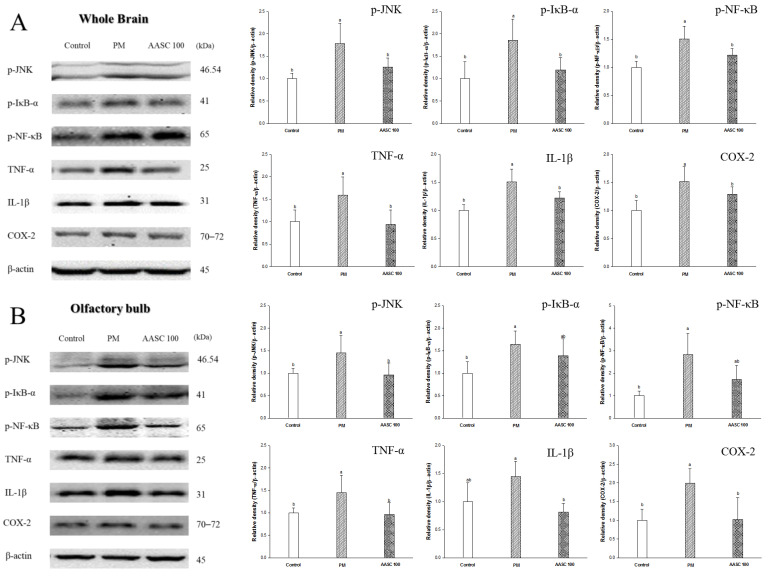
The effect of AASC on proteins related to inflammation in the brains of mice exposed to PM_2.5_. (**A**) Whole brain and (**B**) olfactory bulb. Results are indicated as mean ± SD (*n* = 8). Different small letters represent the statistical difference (*p* < 0.05) of each group in a high order.

**Figure 6:**
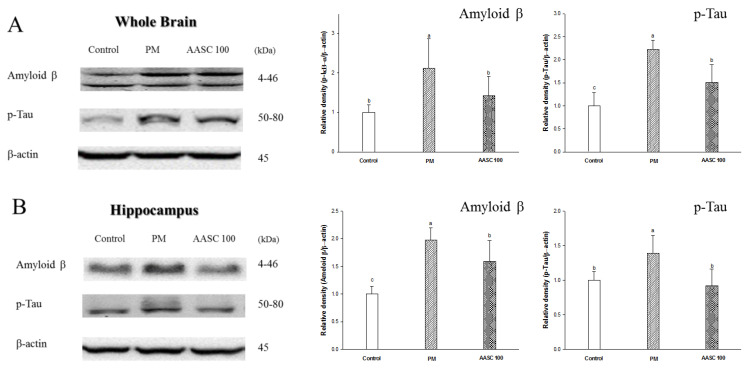
The effect of AASC on amyloid beta and phosphorylated Tau in the brains of mice exposed to PM_2.5_. (**A**) Whole brain and (**B**) olfactory bulb. Results are indicated as mean ± SD (*n* = 8). Different small letters represent the statistical difference (*p* < 0.05) of each group in a high order.

**Figure 7:**
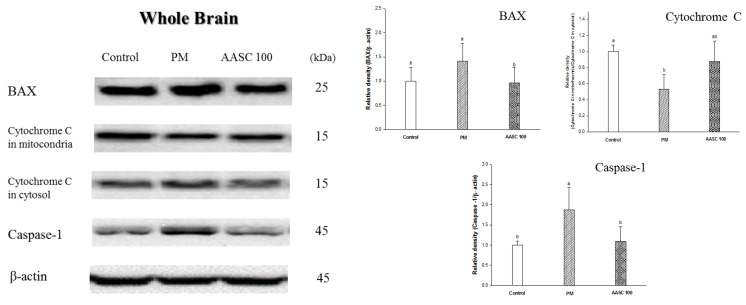
The effect of AASC on proteins related to apoptosis in the brains of mice exposed to PM_2.5_. Results are indicated as mean ± SD *(n* = 8). Different small letters represent the statistical difference (*p* < 0.05) of each group in a high order.

PM_2.5_ directly or indirectly induces neuroinflammation in the brain by producing pro-inflammatory cytokines in the brain and tissue [3,4]. Accordingly, it was confirmed that inflammation was increased in the whole-brain tissue of the PM group compared with the control group (Figure 5A). It is presumed that the increase in lung inflammation confirmed in the previous results also indirectly increased the inflammation of the brain because the pro-inflammatory cytokines can be delivered to the brain tissue through systemic circulation. In addition, an increase in inflammation was also observed in the olfactory bulb of the PM group (Figure 5B). The olfactory bulb is a neural structure of the vertebrate forebrain involved in olfaction, the sense of smell. PM_2.5_ penetrates into the brain through the olfactory bulb, and PM_2.5_ increases inflammation in the olfactory bulb [3]. Neuroinflammation is observed in the earliest stages of cognitive impairment, and the neuronal toxicity associated with inflammation acts as a potential risk factor in the pathogenesis of cognitive impairment [48]. In other words, neuroinflammation plays a prominent role in the pathogenesis of cognitive impairment. Interestingly, this increase in brain inflammation may also be related to cholinergic dysfunction. ACh modulates the production of multiple inflammatory cytokines in the brain through the cholinergic anti-inflammatory pathway, which inhibits pro-inflammatory cytokine release. [49,50]. Therefore, it is speculated that the cholinergic dysfunction confirmed in the previous results may affect increasing brain inflammation (Table 2, Figure 3). The increased inflammatory cytokines in the brain cause various changes in brain tissue. According to the research of Alasmari et al., neuroinflammatory cytokines increase the expression of an amyloid-β pre-cursor protein (APP) and decrease the transport of Aβ into the vascular system, and accordingly could cause an accumulation of Aβ in the brain [51]. In addition, it has been reported that oxidative stress contributes to Aβ generation by inducing the activity of β- and γ-secretases [52]. Another interesting fact is that Aβ is also associated with cholinergic dysfunction. Previous studies have indicated a close relationship between Aβ accumulation and cholinergic dysfunction; Aβ enhances AChE activity and Aβ suppresses the synthesis and release of ACh, interferes with cholinergic receptor signaling, and causes a decrease in the number of cholinergic neurons [24]. In addition, the increased Aβ induces tau phosphorylation and neuronal apoptosis [53,54]. Accordingly, an increase in Aβ expression, tau phosphorylation, and proteins related to neuronal apoptosis were observed in the whole-brain tissue of the PM group (Figure 6A,B). Aβ induces neuronal apoptosis via a mechanism that involves the JNK pathway. JNK is activated through the phosphorylation of JNK at threonine and tyrosine residues, and phosphorylation of JNK was observed in cortical neurons exposed to Aβ [55]. The phosphorylated JNK promotes the accumulation of active apoptosis regulator Bcl 2 Associated X (Bax) in the mitochondria. In addition, JNK is essential for increasing Bax expression. The increased expression of Bax in the mitochondria promotes the release of cytochrome c, which triggers the activation of a caspase cascade [55]. Accordingly, increased BAX expression and cytochrome c release from mitochondria in the whole brain of the PM group was observed (Figure 7). The increase in Aβ and p-Tau in the hippocampus of the PM group is noteworthy (Figure 6B). The hippocampus is an important part related to learning and memory. Aβ causes synaptic deficits in the hippocampus, from the structural to the functional level; accordingly, it leads to impaired synaptic function [56]. In summary, PM_2.5_-induced neuroinflammation and oxidative stress significantly increase the production of Aβ in the brain, and then cause cognitive impairment due to disruption of the cholinergic system. However, it was confirmed that AASC suppressed changes in protein expression in the brain tissue caused by PM_2.5_. These results are also related to the suppression of the increase in inflammation in lung tissue. Ultimately, these results prove that AASC improves cognitive impairment caused by PM_2.5_ through protecting the brain from inflammation and oxidative stress. Moreover, these effects may be related to diCQAs and Q3G, which are the main compounds of AASC.

## 3. Materials and Methods

### 3.1. Sample Preparation 

#### 3.1.1. Sample Extraction

*A. argyi* registered in a local-specific resource in the Korean Forest Service variety protection registration (No. 42, 27 September 2013) was provided by the Namhae Agricultural Association Corporation (Namhae, Korea) [57]. *S. chinesis* was cultivated in Yeongju (Korea) and purchased from duson-aeyagcho (Yeongcheon, Korea). *A. argyi* and *S. chinesis* were ground and then extracted with distilled water at 40 °C for 2 h. After *A. argyi* and *S. chinesis* were evaporated using a vacuum rotary evaporator, those samples were lyophilized using a vacuum freeze drier (N-N series, Eyela Co., Tokyo, Japan). A mixture of *A. argyi* and *S. chinesis* (AASC) was prepared by dissolving *A. argyi* and *S. chinesis* in drinking water at concentrations of 100 mg/kg B.W. and 50 mg/kg B.W., respectively, and mixing them at a ratio of 1:1.

#### 3.1.2. Main Compounds Analysis Using UPLC/Q-TOF-MS/MS

The AASC was dissolved in methanol and then injected into an Acquity UPLC BEH C_18_ column (2.1 × 100 mm, 1.7 μm particle size) equipped with the UPLC system (Waters, Milford, MA, USA) at a flow rate of 0.4 mL/min. The gradient conditions were applied using solvent A (distilled water containing 0.1% formic acid) and solvent B (acetonitrile containing 0.1% formic acid): 1% B/99% A at 0–1 min, 1–100% B at 11.5 min. The electrospray ionization (ESI) conditions are as follows: negative-ion mode, drying gas (N_2_) heated to 120 °C, and collision energy at 20–40 V.

### 3.2. Animal Experimental Design

Four-week-old male Balb/c mice were obtained from a supplier (Samtako, Osan, Korea). All procedures were conducted in accordance with the Institutional Animal Care and Use Committee of Gyeongsang National University (certificate: GNU-200302-M0007, approved on 2 March 2020). The mice were housed at two or three mice per cage (12 h light–dark cycle, 55% relative humidity, and 25 °C) with free access to food and water *ad libitum*, and the mice were randomly divided into four groups (*n* = 9). The mice were exposed to PM_2.5_ at a 500 µg/m^3^ concentration in the whole-body exposure chamber (5 h/day and 5 days/week for 12 weeks). The PM group was a PM_2.5_ exposure group, and the control group was a group that was not exposed to PM_2.5_. The AASC was intragastrically ingested at 50 (AASC 50) and 100 (AASC 100) mg/kg body weight with PM_2.5_ exposure for 12 weeks. At the end of the feeding period, all mice fasted for 12 h, and the mice were sacrificed by CO_2_ inhalation. Blood samples were collected from the abdominal aorta and collected tissues were rinsed with phosphate-buffered saline (PBS) buffer and immediately stored at −70 °C until use.

### 3.3. Behavioral Tests

#### 3.3.1. Y-Maze Test

A Y-maze test was performed using the Smart 3.0 video tracking system (Panlab, Barcelona, Spain) for 8 min [58].

#### 3.3.2. Morris Water Maze Test

A Morris water maze test was performed for 6 days (5 days training, 1-day test) in the pool filled with opaque water using melted squid ink (Cebesa, Valencia, Spain). This test was also observed using the Smart 3.0 video tracking system [20].

### 3.4. Cholinergic System

#### 3.4.1. Tissue Pretreatment

After homogenizing the brain with PBS, the supernatant was obtained by centrifugation (14,000× *g* for 30 min at 4 °C). The supernatants were used for ACh and AChE measurements. 

#### 3.4.2. ACh Content

Alkaline hydroxylamine reagent was added to the supernatant, and then 0.37 M FeCl_3_ in 0.1 N HCl and 0.5 N HCl were added after 1 min. Finally, the absorbance was measured at 540 nm [59]. 

#### 3.4.3. AChE Activity

A mixture of 50 mM sodium phosphate buffer (pH 8.0) and the supernatant were incubated at 37 °C for 15 min. Then, the Ellman’s reaction mixture was added to mixture, and absorbance was measured at 405 nm after incubation at 37 °C (20 min) [60].

### 3.5. Serum Biomarkers Analysis

#### 3.5.1. Serum LDH Levels

Blood samples were centrifuged at 10,000× *g* for 10 min at 4 °C and then the supernatant (serum) was used. The concentration of serum LDH was analyzed with a clinical chemistry analyzer (Fuji Dri-Chem 4000i; Fuji Film Co., Tokyo, Japan). 

#### 3.5.2. Serum FRAP Activity

The FRAP assay was developed by Benzie and Strain [61]. The serum was reacted with the FRAP reagent. After incubation for 30 min in the dark, absorbance was measured at 593 nm (Epoch2, BioTek, Winooski, VT, USA).

### 3.6. Oxidative Stress and Mitochondrial Function

#### 3.6.1. Tissue Pretreatment

Tissues were homogenized with 10 volumes of PBS for the measurement of MDA and SOD levels. Mitochondria were isolated following the procedure of Dragicevic [62].

#### 3.6.2. Antioxidant System

The homogenates were centrifuged at 400× *g* for 10 min at 4 °C. After removing the supernatant, 1 × cell extraction buffer was added in pellets. The mixtures were incubated on ice for 30 min, and the SOD contents were measured in supernatant obtained from centrifugation (10,000× *g* for 10 min at 4 °C) using a SOD kit.

To measure MDA contents, supernatants were obtained from centrifugation (6000× *g* for 10 min at 4 °C) of homogenates. After 1% phosphoric acid and 0.67% TBA were added to supernatants, it was incubated for 1 h at 95 °C, and then absorbance was measured at 532 nm.

The reduced GSH levels were measured by homogenizing the tissues with phosphate buffer. The supernatant obtained from centrifugation at 10,000× *g* for 15 min was mixed with 5% metaphosphoric acid. After centrifuging this at 2000× *g* for 2 min, 0.65 N NaOH, Tris-HCl buffer, and O-phthalaldehyde were added to the supernatant, and it was left in the dark for 15 min.

The supernatant was mixed with 5% metaphosphoric acid and centrifuged at 2000× *g* for 2 min. Tris-HCl buffer (0.26 M, pH 7.8), 0.65 N NaOH, and O-phthalaldehyde were added to the supernatant and left in the dark for 15 min. The fluorescence was measured using an Infinite 200 (Tecan Co., San Jose, CA, USA) (excitation: 320 nm, emission: 420 nm) [63].

#### 3.6.3. Mitochondria Function

The isolated mitochondria were mixed with 25 μM DCF-DA and KCl-based respiration buffer and then incubated for 20 min to measure the ROS at fluorescence (excitation filter 485 nm and emission filter 528 nm) using a fluorometer (Infinite 200, Tecan Co., San Jose, CA, USA). 

To measure MMP, 1 μM JC-1 was added to isolated mitochondria and incubated for 20 min. Then, fluorescence (excitation: 530 nm, emission: 590 nm) was measured using a fluorometer. 

The ATP was analyzed using an ATP bioluminescence assay kit (Promega, Madison, WI, USA) using a luminescence meter (Promega, Madison, WI, USA).

### 3.7. Western Blot Analysis

#### 3.7.1. Protein Extraction

Tissues of mice were homogenized in lysate buffer, and 1% protease inhibitor cocktails were added. After the homogenate was centrifuged at 13,000× *g* for 10 min at 4 °C, the supernatants were used in the experiment. 

#### 3.7.2. Western Blotting

The protein samples were separated by SDS- polyacrylamide gel electrophoresis (PAGE) and electro-transferred to a polyvinylidene difluoride (PVDF) membrane. The membranes were incubated in primary antibodies (Supplementary Data, Table S1) diluted in TBST overnight at 4 °C. After washing, the membranes were incubated with a secondary antibody solution for 1 h, and then treated with the chemiluminescence reagent to the membrane. The bands were detected using the iBright™ CL1000 Imaging System (Thermo Fisher, Waltham, MA, USA). 

### 3.8. Statistical Analysis 

Data are represented as mean ± SD. Statistical analysis was performed with one-way analysis of variance (ANOVA) followed by Duncan’s multiple range test using the SAS program (Ver. 9.4 SAS Institute, Cary, NC, USA). The different small letters represent the statistical differences (*p* < 0.05) of each group in high order.

## 4. Conclusions

Long-term exposure to PM_2.5_ causes oxidative stress, mitochondrial dysfunction, and inflammatory responses in the lung and brain tissues. Lung damage affects the increase in inflammatory cytokines in the brain and the increase in inflammation, as well as oxidative stress, in brain tissue, and promotes the formation of amyloid β, which in turn induces cholinergic dysfunction, phosphorylation of tau and apoptosis activation, leading to cognitive impairment. However, AASC inhibited these changes to brain tissue caused by PM_2.5_ by suppressing the increase in oxidative stress and inflammation in both lung and brain tissues. This effect is presumed to be related to polyphenol compounds in AASC. In other words, these results suggest that AASC, containing substances with potent antioxidant and anti-inflammatory activities, can be a preventive strategy to protect against brain damage caused by PM_2.5_. Therefore, this study suggested the possibility of research on mitigating PM_2.5_ toxicity through plant resources. 

## Figures and Tables

**Table 1:** The identified compounds in the AASC.

No.	RT (min)	Parent Ion (*m*/*z*)	MS^2^ Fragments (*m*/*z*)	Proposed Compound
1	0.77	192.05	191	Quinic acid
2	2.83	354.08	191, 135	Chlorogenic acid
3	2.97	564.14	563, 473, 443, 383, 353	Isoschaftoside
4	3.12	610.15	609, 301, 300	Rutin
5	3.21	478.07	301, 151	Quercetin-3-gucuronide
6	3.29	516.12	353, 191, 179,173, 135	3,4-dicaffeoylquinic acid
7	3.35	516.12	191, 179	3,5-dicaffeoylquinic acid
8	3.4	516.12	191, 179, 135, 353	4,5-dicaffeoylquinic acid

**Table 2:** Effect of AASC on the cholinergic system in serum of mice exposed to PM_2.5_.

	Control	PM	AASC 50 *	AASC 100 *
ACh contents (mmole/mg of protein)	2.18 ± 0.17 ^a^	1.68 ± 0.08 ^c^	1.95 ± 0.10 ^b^	1.96 ± 0.10 ^b^
AChE activity (% of control)	100.0 ± 7.0 ^b^	108.5 ± 4.1 ^a^	89.2 ± 5.3 ^c^	89.7 ± 2.7 ^c^

Results are indicated as mean ± SD (*n* = 9). Different small letters represent the statistical difference (*p* < 0.05) of each group in a high order. * The number indicates dietary concentration (mg/kg body weight) of AASC.

**Table 3:** Effect of AASC on biochemicals related to oxidative stress in the brain and lung tissues of mice exposed to PM_2.5_.

	Brain				Lung			
	Control	PM	AASC 50 *	AASC 100 *	Control	PM	AASC 50 *	AASC 100 *
MDA contents (nmole/mg of protein)	3.50 ± 0.48 ^bc^	4.32 ± 0.46 ^a^	3.79 ± 0.15 ^ab^	3.04 ± 0.07 ^c^	1.34 ± 0.04 ^b^	1.47 ± 0.05 ^a^	1.30 ± 0.10 ^b^	1.31 ± 0.08 ^b^
SOD contents (U/mg of protein)	4.10 ± 0.31 ^a^	3.36 ± 0.16 ^b^	3.86 ± 0.20 ^a^	3.73 ± 0.15 ^a^	5.14 ± 0.41 ^a^	4.17 ± 0.17 ^b^	4.67 ± 0.39 ^ab^	4.63 ± 0.31 ^ab^
Reduced Glutathione (% of control)	100.0 ± 8.7 ^a^	74.9 ± 12.3 ^b^	85.2 ± 8.9 ^ab^	107.1 ± 14.4 ^a^	100.0 ± 24.0 ^a^	61.0 ± 12.1 ^b^	77.8 ± 8.4 ^ab^	74.8 ± 7.2 ^ab^

Results are indicated as mean ± SD (*n* = 9). Different small letters represent the statistical difference (*p* < 0.05) of each group in a high order. * The number indicates dietary concentration (mg/kg body weight) of AASC.

**Table 4:** Effect of AASC on biomarkers related to oxidative stress in serum of mice exposed to PM_7.5_.

	Control	PM	AASC 50 *	AASC 100 *
FRAP activity (Absorbance at 593 nm)	0.43 ± 0.02 ^a^	0.28 ± 0.01 ^c^	0.31 ± 0.01 ^c^	0.35 ± 0.03 ^b^
LDH contents (U/L)	187.3 ± 23.8 ^ab^	256.0 ± 42.8 ^a^	146.3 ± 33.3 ^b^	186.0 ± 34.4 ^ab^

Results are indicated as mean ± SD (*n* = 9). Different small letters represent the statistical difference (*p* < 0.05) of each group in a high order. * The number indicates dietary concentration (mg/kg body weight) of AASC.

**Table 5:** Effect of AASC on mitochondrial function in brains of mice exposed to PM_2.5_.

	Brain				Lung			
	Control	PM	AASC 50 *	AASC 100 *	Control	PM	AASC 50 *	AASC 100 *
ROS contents (% of control)	100.0 ± 6.4 ^b^	133.7 ± 16.9 ^a^	135.74 ± 15.6 ^a^	71.1 ± 4.6 ^c^	100.0 ± 26.5 ^b^	136.4 ± 25.5 ^a^	112.6 ± 32.6 ^ab^	107.6 ± 20.3 ^ab^
MMP (% of control)	100.0 ± 14.4 ^b^	73.6 ± 12.2 ^c^	101.5 ± 8.8 ^b^	123.2 ± 6.8 ^a^	100.0 ± 14.4 ^bc^	74.1 ± 26.4 ^c^	118.5 ± 31.9 ^ab^	149.7 ± 5.9 ^a^
ATP contents (nmole/mg)	318.5 ± 67.0 ^a^	90.1 ± 16.0 ^b^	79.5 ± 3.0 ^b^	421.0 ± 146.2 ^a^	306.8 ± 21.4 ^a^	170.2 ± 53.9 ^b^	241.1 ± 56.4 ^ab^	310.0 ± 63.2 ^a^

Results are indicated as mean ± SD (*n* = 9). Different small letters represent the statistical difference (*p* < 0.05) of each group in a high order. * The number indicates dietary concentration (mg/kg body weight) of AASC.

## Data Availability

The data underlying this article are shared on reasonable request to the corresponding author.

## Supplementary Materials

The following supporting information can be downloaded at: https://www.mdpi.com/article/10.3390/plants12061230/s1, Figure S1: Effect of *Artemisia argyi* on PM_2.5_-induced cytotoxicity in (A) RPMI2650; nasal cell line (B) A549; alveolar basal epithelial cell line and (C) BV2 cell; microglial cell line and effect of *Saururus chinensis* on PM_2.5_-induced cytotoxicity in (D) RPMI2650, (E) A549, and (F) BV2 cell. Figure S2: Effect of (A) *Artemisia argyi* and (B) *Saururus chinensis* on lipopolysaccharide (LPS)-induced nitroxide (NO) production in BV2; microglial cell line. Figure S3: Analysis of Artemisia argyi, *Saururus chinensis*, AASC and Blank using UPLC/Q-TOF-MS/MS chromatography in negative ion mode. Table S1: List of antibodies and their information used in this study.
