# Supplementary material for: A Mixture of Artemisia argyi and Saururus chinensis Improves PM2.5-Induced Cognitive Dysfunction by Regulating Oxidative Stress and Inflammatory Response in the Lung and Brain
Source: Plants (Basel). 2023 Mar 8;12(6):1230. doi: 10.3390/plants12061230 (PMC10059966; doi:10.3390/plants12061230)
Supplement: Supplementary file 1 [file plants-12-01230-s001.zip › plants-2241911-supplementary.pdf]

Table S1. List of antibodies and their information used in this study

Antibody	Catalog	Conc.	Manufacturer
AChE	sc-373901	1:500	Santa Cruz Biotech (Dallas, TX, United States)
Amyloid β	sc-28365	1:500	Santa Cruz Biotech (Dallas, TX, United States)
BAX	sc-7480	1:500	Santa Cruz Biotech (Dallas, TX, United States)
β-actin	sc-69879	1:500	Santa Cruz Biotech (Dallas, TX, United States)
Caspase-1	sc-392736	1:500	Santa Cruz Biotech (Dallas, TX, United States)
COX-2	sc-376861	1:500	Santa Cruz Biotech (Dallas, TX, United States)
Cytochrome C	sc-13560	1:500	Santa Cruz Biotech (Dallas, TX, United States)
IL-1β	sc-4592	1:500	Santa Cruz Biotech (Dallas, TX, United States)
p-JNK	sc-6254	1:500	Santa Cruz Biotech (Dallas, TX, United States)
p-tau	sc-12952	1:500	Santa Cruz Biotech (Dallas, TX, United States)
p-IκB-α	sc-8404	1:500	Santa Cruz Biotech (Dallas, TX, United States)
p-NF-κB	sc-8008	1:500	Santa Cruz Biotech (Dallas, TX, United States)
TNF-α	sc-133192	1:500	Santa Cruz Biotech (Dallas, TX, United States)
ChAT	20747-1AP	1:1000	Bioneer (Daejeon, Korea)

AChE, acetylcholinesterase; BAX, bcl-2-like protein 4; COX-2, cyclooxygenase-2; IL-1β, interleukin 1 beta; p-JNK, phosphorylated c-Jun N-terminal kinase; p-IκB, phosphorylated nuclear factor of kappa light polypeptide gene enhancer in B-cells inhibitor, alpha; p-NF-κB, phosphorylated nuclear factor kappa-light-chain-enhancer of activated B cellsTNF-α, tumor necrosis factor-alpha; ChAT, choline acetyltransferase

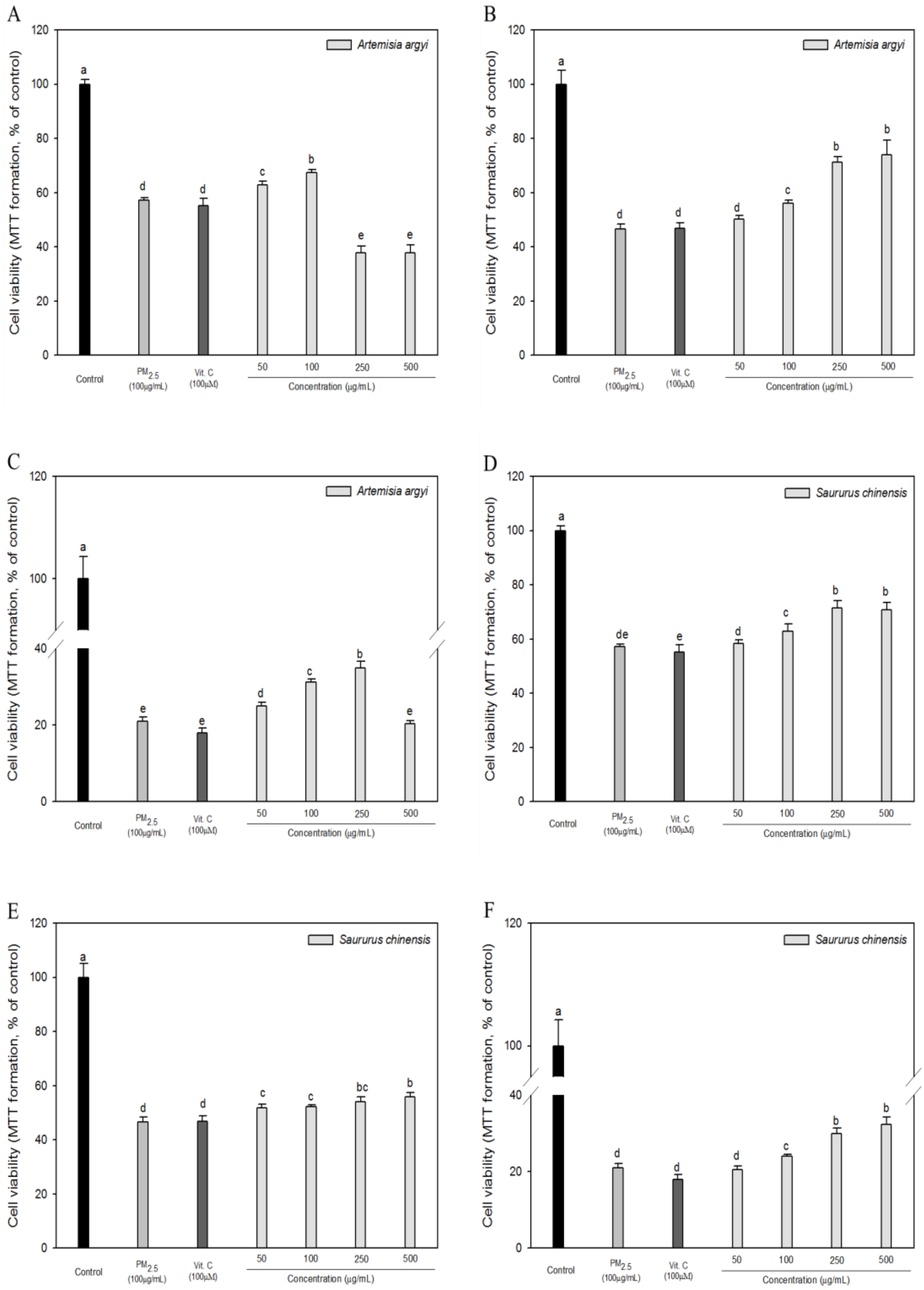

Figure S1. Effect of *Artemisia argyi* on $PM_{2.5}$-induced cytotoxicity in (A) RPMI2650; nasal cell line (B) A549; alveolar basal epithelial cell line and (C) BV2 cell; microglial cell line and effect of *Saururus chinensis* on $PM_{2.5}$-induced cytotoxicity in (D) RPMI2650, (E) A549, and (F) BV2 cell. Results are indicated as mean ± SD (n = 3). Different small letters represent statistical difference ($p < 0.05$) of each group in a high order.

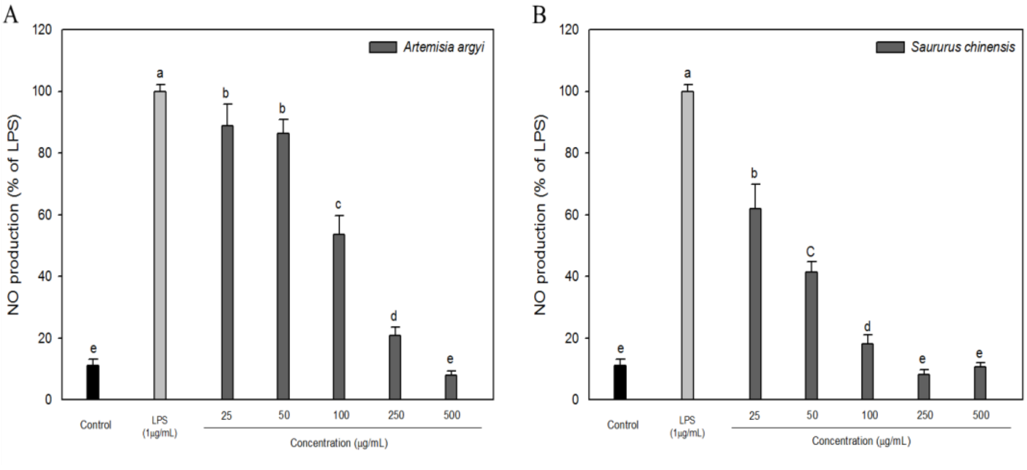

Figure S2. Effect of (A) *Artemisia argyi* and (B) *Saururus chinensis* on lipopolysaccharide (LPS)-induced nitroxide (NO) production in BV2; microglial cell line. Results are indicated as mean ± SD (n = 3). Different small letters represent statistical difference ($p < 0.05$) of each group in a high order.

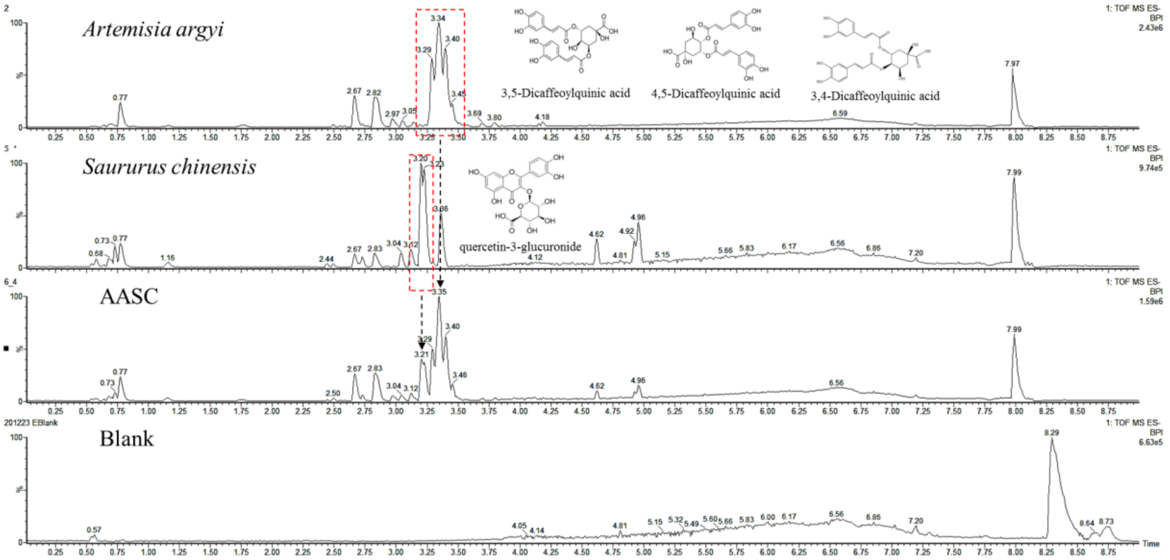

Figure S3. Analysis of *Artemisia argyi, Saururus chinensis,* AASC and Blank using UPLC/Q-TOF-MS/MS chromatography in negative ion mode.
